# The Cost of Managing Moderate Wasting Using Local Foods: Evidence from Three Interventions in Northeast Nigeria

**DOI:** 10.1017/S1368980025101213

**Published:** 2025-09-30

**Authors:** Stacie Gobin, Margaret Holmesheoran, Pauline Adah, Halima Haruna, Amanda Yourchuck, Chloe Puett

**Affiliations:** 1 Gobin Global, 1 Page Ave, Unit 280, Asheville, NC 28139, USA; 2 USAID, 1300 Pennsylvania Avenue NW, Washington, DC 20004, USA; 3 USAID Advancing Nutrition, 2733 Crystal Drive, 4th Floor, Arlington, VA 22202, USA; 4 Action Against Hunger, 3 Adamu Ciroma Cres, Jabi, Abuja 900288, Federal Capital Territory, Nigeria; 5 Stony Brook University, Department of Family, Population and Preventive Medicine, Program in Public Health; Level 3, Room 071, Health Sciences Center, Stony Brook, NY 11794-8338, USA

**Keywords:** Malnutrition, Costs and cost analysis, Supplementary feeding, Access to healthy food, Community, Nigeria

## Abstract

**Objective:**

Management of moderate wasting (MW) is an important component of country-level strategies to address wasting, given high caseloads and susceptibility to illness and death. However, many countries experience challenges in providing targeted supplementary feeding programmes with specially formulated foods involved in managing MW. Some implementing agencies have developed a community-based programme using locally available foods (LF) for MW management known as Tom Brown. This study assessed the costs and cost-efficiency of three Tom Brown programmes (two with an 8-week supplementation duration, one with a 10-week duration).

**Design:**

We assessed institutional costs and selected estimates of societal costs to households and community volunteers.

**Setting:**

Northeast Nigeria

**Participants:**

Programme staff

**Results:**

Total cost per child ranged from $155 to $184 per 8-week programme and $493 per 10-week programme. Monthly LF supplementation cost per child ranged from $5 to $21. Unit costs were influenced by implementation duration and variations in programme features including storage and transportation models, the inclusion of voucher transfers and volunteer cadre models. Opportunity costs to beneficiaries and volunteers in preparing recipes were substantial. Cash/voucher components, when used, represented a cost driver for institutional and societal costs.

**Conclusions:**

An updated WHO guideline emphasises the role of LF for supplementing MW children who lack other risk factors. Given that specially formulated foods are not necessary for all MW children to recover, programme approaches using LF are important options for managing MW. This study from Nigeria provides the first cost estimates for using LF to manage MW. Future research is needed on the effectiveness and cost-effectiveness of these approaches.

Management of moderate wasting is an important component of country-level strategies to address wasting, given high numbers of moderately wasted children and their susceptibility to illness and death^(1)^. For over two decades, community-based management of acute malnutrition approaches have reduced morbidity and mortality using targeted supplementary feeding programmes (TSFP) that supplement moderately wasted children using specially formulated foods (SFF) such as ready-to-use supplementary foods and fortified blended flours. While these products provide the micro- and macronutrients needed for rehabilitation from moderate wasting, many programme implementation partners cannot support the continual procurement and supply chain management of SFF, resulting in a lack of availability and limited accessibility that can inhibit programme effectiveness. SFF are typically unavailable in local markets, so there are few alternatives if caregivers cannot obtain them through routine TSFP.

In light of challenges related to the availability of SFF, implementing partners in some countries, including Nigeria, have developed programmatic approaches using locally available foods for the management of moderate wasting. The specific locally available food ration provided differs across programmes. A recent review outlined that many locally available food programmes are designed around local preparation of a flour adhering to nutrient ratios, with, for example, a locally available plant-based protein and animal-based protein being included, as a ratio, at twice the quantity of a locally available carbohydrate ingredient (e.g. maize, millet or sorghum)^(2)^. Other programmes provide some local food rations and recipes and additionally offer food vouchers to participants for purchasing ingredients locally.

In 2023, the WHO released an updated guideline on the prevention and management of wasting and nutritional oedema (acute malnutrition) in infants and children under 5 years of age^(3)^. In this guideline, factors that place some moderately wasted children at higher risk of mortality are discussed, along with recommendations that these children be prioritised to receive SFF through the health system. This guideline further recommends that moderately wasted children not meeting one or more of these risk factors can be supplemented using locally available food-based approaches. However, the evidence base is still growing related to the effectiveness of these approaches^(3)^. Of the ten peer-reviewed studies identified that were related to the use of locally available foods for managing moderate wasting, five reported recovery rates^(4–8)^. All met Sphere standards for recovery^(9)^. Some studies also compared locally available foods with other commercially produced products like corn-soya blend/plus; these studies found locally available foods to be non-inferior, or not unacceptably worse than standard treatment with SFF such as corn-soya blend^(7,10)^.

More empirical data are needed on these approaches to inform their potential scale-up, including replicability in other contexts. An important aspect of scalability and replicability is cost. Analysis and documentation of programme costs help implementers, governments and funders in decision-making and priority setting. However, existing cost data for managing moderate wasting represent programme approaches using SFF distributed through local health centres rather than interventions using locally available foods prepared and/or distributed at the community level. For example, existing cost-efficiency data from Sierra Leone found the cost per child enrolled in TSFP to be $83–87, depending on the supplement used (e.g. ready-to-use supplementary foods or various fortified blended flours) (supplements provided for 12 weeks; values in 2018 USD)^(11)^. A study in Mali reported a range of costs per child enrolled of $89 from ready-to-use supplementary foods to $100 for the distribution of a locally milled flour mixture (supplements provided weekly for 4 weeks and biweekly thereafter for 12 weeks; 2015 USD)^(12)^. Finally, a study from Indonesia of daily and weekly distribution of locally produced ready-to-use biscuits for rehabilitating moderately and mildly wasted children reported costs per beneficiary enrolled of $376 for daily distribution and $332 for weekly distribution (supplementation duration varied: daily supplementation provided for 56 days average and weekly supplementation for 8·5 weeks average; 2007 USD)^(4)^.

In the context of limited evidence on the management of moderate wasting using locally available foods, Nigeria’s use of locally available foods for the management of moderate wasting offers an opportunity to conduct a costing study to inform future implementation and potential scale-up of these approaches.

The objective of this costing study was to document the costs for a community-based supplementary feeding programme using local foods for the management of moderate wasting, known as Tom Brown, as implemented by three partners in Northeast Nigeria. Having data on the costs of these approaches will assist local implementers and stakeholders with programme planning and provide evidence to the global nutrition community to assess the scalability and replicability of these approaches in diverse settings, particularly in low-resource environments facing similar nutritional and logistical challenges.

This study aims to contribute to the global evidence base on the cost of the management of moderate wasting, particularly for approaches using locally available foods. This study also aims to provide findings and implications in light of the recently released WHO guideline, which includes guidance on moderate wasting for the first time and emphasises the use of local and family foods for nutritional support. Given the potential of locally available food-based approaches like Tom Brown to offer feasible alternatives to TSFP in contexts where SFF are unavailable, data on the costs of these approaches will be an important factor in determining their feasibility and scalability. Thus, our findings will provide important considerations for governments, non-governmental organisations and funders aiming to integrate local food-based strategies into broader nutrition programmes globally.

## Methods

### Geographic context

In Nigeria, 11·6 % of children aged 6–59 months are nutritionally wasted^(13)^. Northeast Nigeria is one of the most affected regions of the country, with an estimated 1·5 million wasted children living in the three most affected states of Adamawa, Borno and Yobe, of which 1·02 million were moderately wasted^(14)^. Additionally, an estimated 207 000 pregnant and lactating women experienced wasting and were in need of nutrition interventions during 2022^(14)^. In Borno state, the geographic focus of this study, the most recent estimates show a child wasting prevalence of 14·3 % up to 18·1 % in some areas^(15)^. Despite the significant need, supplementation coverage for moderate wasting is inadequate, with about 70 % of moderately wasted children across the northeast not receiving support^(16)^. Despite there being moderately wasted children present across the country, coverage of standard TSFP is concentrated in the northeast and is insufficient to meet needs.

### Programmatic context

We examined the cost of Tom Brown as implemented by three partners: Catholic Relief Services (CRS), Premiere Urgence Internationale (PUI) and Save the Children International (SCI). Table 1 provides a summary of the programmes. Additional programmatic details are reported in the online Supplemental Materials.

**Table 1.** Programme features

	CRS	PUI	SCI
Period analysed	June 2021–April 2023	January 2021–June 2023	June 2021–April 2023
Programme structure	CRS and two local partners	PUI and no local partners	SCI and one local partner
Programme setting	Emergency/rural + peri-urban	Emergency/rural	Emergency/rural
Eligibility and selection criteria	Caregivers of MW children 6–59 months enrolled to participate in the production of SFF for children, weekly meetings with nutrition education and food production		
Cash/voucher transfers	Yes, in the peri-urban model	No	No
Food storage model	Procured in batches, stored in satellite offices and delivered to LM houses weekly. The peri-urban model has moved to cash vouchers.	Procured in batches and stored in a central WFP storage facility in Monguno (at no cost to the programme) and delivered to LM houses weekly.	Procured and stored centrally at the SCI Maiduguri office/warehouse and delivered to LM houses weekly.
Group facilitator (volunteer role)	Lead mother	Lead mother	Lead mother
Children/beneficiaries enrolled	12 890	1920	3376
Active groups	1081	160	315
Cycle duration	8 weeks	8 weeks	10 weeks

CRS, Catholic Relief Services; FSL, food security and livelihoods; LM, lead mother; MW, moderate wasting; PUI, Premiere Urgence Internationale; SCI, Save the Children International; SFF, specially formulated food; WFP, World Food Programme.

#### Catholic Relief Services

In the Tom Brown approach, developed by CRS, caregivers of children aged 6–59 months with moderate wasting (mid-upper-arm circumference (MUAC) ≥ 115 mm to < 125 mm) were enrolled in groups whose purpose was to participate actively in the production of the supplementary food for their children throughout the implementation period of 8–10 weeks^(17)^. These groups of women were trained and supported by community volunteers, including community nutrition mobilisers, lead mothers, field assistants and others, to produce the Tom Brown flour made from locally-sourced ingredients including millet, maize or sorghum, soya and groundnuts. The flour is taken home and prepared as a porridge for their children. During implementation, groups of women gather weekly to produce Tom Brown and participate in nutrition education for 8–10 weeks.

The CRS Tom Brown approach follows their Tom Brown Implementation Guidelines and uses a 6:3:1 ratio of the following ingredients, respectively: cereals (maize, millet and/or sorghum), soya and groundnuts^(17)^. Each enrolled child received 1·5 kg of the Tom Brown flour on a weekly basis. Caregivers are instructed to provide enrolled children with approximately 214 g of the flour prepared as a porridge per day, in two to three servings, in addition to their usual meals. Children’s MUAC is monitored on a weekly basis throughout the duration of the programme, and children who deteriorate into severe wasting are referred for treatment through outpatient or inpatient services, as appropriate. Although MUAC is monitored, children are retained in the programme for the full period, even if they achieve a healthy MUAC (MUAC ≥ 125 mm) before the end of the programme. In peri-urban areas, a cash and voucher approach is used whereby the lead mother and two assistant beneficiary mothers purchase local food items from vendors. In rural areas, CRS procures food ingredients in bulk and stores them for a maximum of four weeks in their warehouse and field-based satellite houses. They deliver the food ingredients to the lead mothers’ households for weekly storage (depending on the location, some deliveries on a different schedule are stored in the household for slightly longer). The groups follow a 7-day schedule, with 3 days used for the food preparation, after which the flour is taken home (each group selects a schedule that works best for them). The schedule is followed for an 8-week session. During the 8-week session, a group of twelve caregivers convenes at the lead mother’s home for flour preparation and also takes part in a weekly nutrition education session. Each lead mother is also responsible for receiving and retaining a non-food item (NFI) programme kit on behalf of her group. The kits typically include cooking utensils, storage containers, a floor mat, MUAC tapes and basic hygiene materials.

The flour preparation process lasts 3 days: Day 1: provision of grains, clean-up and washing; Day 2: drying the grains, soaking and deshelling of the soya beans; and Day 3: grinding the grains, porridge preparation and flour distribution.

#### Premiere Urgence Internationale

PUI uses the same Tom Brown ingredients as provided in the CRS Tom Brown Implementation Guidelines, following the 6:3:1 ratio, but provides the groups with slightly higher amounts of each food item. Weekly portion guidance and MUAC monitoring are the same as in the CRS programme and as per the CRS Tom Brown Implementation Guidelines. PUI procures the food ingredients from local food vendors and stores them in a central World Food Programme storage facility in Monguno at no financial cost to the programme (the economic cost of this storage was estimated for the purposes of this analysis). Ingredients are purchased in bulk for each Tom Brown cohort from local food vendors near the warehouse in Monguno. For delivery to the lead mothers’ homes, a request is submitted from PUI to the World Food Programme for the release of food ingredients from the warehouse. PUI staff pick up the ingredients and transport them to the lead mothers’ homes on a weekly basis. The groups follow a 7-day schedule, with 3 days used for food preparation, after which the flour is taken home. Like CRS, PUI’s Tom Brown sessions last for 8 weeks. Each group receives a basic NFI kit similar to the one provided by CRS, which the lead mother is responsible for taking care of and keeping safe during the programme duration.

The steps of PUI’s Tom Brown approach include screening of children for moderate wasting by community nutrition mobilisers, selection of lead mothers, weekly refresher training of lead mothers and preparation and distribution of flour. In addition, community nutrition mobilisers provide infant and young child feeding and hygiene counselling to lead mothers and other beneficiary mothers in the groups and take weekly MUAC measurements.

The flour preparation process lasts 3 days: Day 1: provision of grains, clean-up and washing, soaking the soya beans and cereals; Day 2: dehusk the beans, drying the grains; and Day 3: roasting and drying the soya beans, lightly roasting the sorghum and millet, mixing the ingredients, grinding the grains, preparing the porridge and distributing the flour.

#### Save the Children International

SCI uses the same Tom Brown recipe as provided in the CRS Tom Brown Implementation Guidelines, following the 6:3:1 ratio. Weekly ration size, portion guidance and MUAC monitoring are the same as in the CRS programme and as per the CRS Tom Brown Implementation Guidelines. Participating mothers are also provided with infant and young child feeding messaging and are facilitated through an assessment of challenges and root causes of malnutrition in the household (i.e. hygiene, breastfeeding difficulties, etc.). Tom Brown ingredients are procured by SCI and stored at the central Maiduguri office/warehouse. They are delivered to Tom Brown groups on a weekly basis to avoid issues with storage at the site (lead mother’s house) and issues with food ingredients. SCI works with a local partner, Green Code, for the procurement of the grains, delivery to the community on a weekly basis, enrolment through field assistants and supervision of groups by nutrition officers. The groups follow a 7-day schedule, with 4 days used for food preparation, after which the flour is taken home. SCI implements Tom Brown on a 10-week cycle, which is 2 weeks longer than the CRS and PUI programmes. During the 10-week period, a group of 6–12 caregivers convene at the lead mother’s home for weekly flour preparation. Each group receives a basic NFI kit similar to the one provided by CRS.

The steps of SCI’s Tom Brown approach include active case finding by community nutrition mobilisers, identification of a group facilitator or lead mother and the production of the Tom Brown flour. Counselling and materials are also provided to the lead mothers, including the NFI, which they were responsible for keeping safe and in good condition throughout the entire programme cycle. The food preparation process lasts 4 days: Day 1: provision of grains, clean-up and washing; Day 2: drying the grains, soaking and deshelling of the soya beans; Day 3: drying all the grains; and Day 4: grinding the grains, porridge preparation and flour distribution.

Programmes implemented in more dispersed rural areas may face higher logistical and transportation costs compared with those in peri-urban or clustered localities.

### Cost-efficiency analysis

This analysis focuses on estimating cost-efficiency by calculating the *unit cost per child per programme.* As cost data were analysed from three different partners using slightly different programme designs and delivery structures for Tom Brown, we were able to assess how differences in implementation affected the cost per child enrolled in the programme. Each child enrolled represents a single programme recipient. Each of the partners collected programme monitoring data on the number of participating mothers, but only CRS and SCI directly tracked the number of moderately wasted children enrolled and registered in the Tom Brown programme (and the dosage of food provided to each household was based on the number of children). PUI, on the other hand, tracked the number of participating mothers with an estimated 1:1 ratio of mothers to children. In other words, PUI provided food based on the assumption that there was only one moderately wasted child per enrolled and registered mother. Annualised and monthly costs allow for a standardised comparison across programmes with differing durations.

There were several reasons that cost-efficiency analysis was chosen for this study over cost-effectiveness analysis, which estimates costs based on the number of beneficiaries recovered. First, investment and programme planning decisions, which this study sought to inform, are based on the number of children enrolled (used in cost-efficiency analysis), not the number of children recovered (used in cost-effectiveness analysis). Second, the quality of the programme outcome data, which is required for the cost-effectiveness analysis, could not be verified for these particular approaches. The design of this costing exercise did not include primary data collection on programme outcomes; instead, it relied on existing outcome data, as reported by the programmes’ monitoring systems. An early review of admissions and recovery data showed that recovery rates were 96–99 %, which are not only difficult to externally verify as part of the costing study but are very high compared with other similar programmes in, for example, Mali and Sierra Leone, which have recovery rates ranging between 57 and 70 %^(11,12)^. Finally, with recovery rates between 96 and 99 %, if assumed to be accurate, cost-efficiency estimates of cost per child enrolled would be nearly the same as cost-effectiveness estimates of cost per child recovered (since nearly all enrolled children are reported to have recovered).

### Cost compilation and considerations

This study focused on institutional costs, including all relevant inputs required for programme implementation. Institutional costs only provide information on the costs borne by implementing agencies, and do not address the societal costs or the time and money spent by participants and communities in making a programme function effectively. Therefore, the institutional cost estimates were supplemented with estimates of societal costs. These were based on local resources or infrastructure that were needed to implement these approaches. While time limitations prohibited a full societal costing, we estimated specific ‘ingredients,’ cost calculations for activities described as time-intensive in programme documentation or inputs that influenced programme scalability.

Institutional cost data for programme implementation were collected from partners and analysed using step-down cost accounting^(18)^. This approach included reviewing outputs from each partner’s accounting databases during the same time period as enrolment data to develop estimates of total programme costs. Using programme expenditure data, the step-down cost accounting method allocated the cost of support departments (e.g. management, accounting) and technical departments (e.g. food security and livelihoods (FSL)) in cases where these costs were not directly charged to the Tom Brown programme but contributed to its implementation.

For estimating the monetary value of programme recipient time spent on the programme, we have used an estimate of the most relevant local daily wage as a shadow wage for programme beneficiaries in the calculations. A shadow wage is an estimation of the economic value of the resource when the direct measurement of the market value is unavailable. For volunteer roles (e.g. lead mother, field assistant and in some cases community nutrition mobilisers), we assumed the published national minimum wage^(19)^, and for government staff, where relevant, we assumed the equivalent of a mid-range field-based Ministry of Health supervisor.

Time allocation interviews were conducted with technical and support staff to allocate staff costs and other costs that are shared between programmes and enable general programme functioning. Implementing staff included those from nutrition and FSL teams since the Tom Brown approaches were, in some cases, multisectoral due to a voucher/cash transfer component. Questions accounting for staff time spent on supporting the nutrition and FSL components of the programme were included. Where programme staff were unable to provide specific proportions of individual staff time, estimates were provided for the entire shared costs relative to the nutrition- and FSL-specific technical components. This information assisted in allocating to the programme all staff and support costs and apportioning other non-programme-specific support costs, such as monitoring, evaluation, accountability and learning (MEAL) and logistics.

In the CRS Tom Brown programme, where cash/vouchers were used in peri-urban areas, we isolated these components to assess their specific cost implications relative to direct procurement models used in the PUI and SCI programmes.

While all three partners implemented the approaches in a rural emergency setting, CRS also implemented them in a peri-urban setting in Maiduguri; thus, direct costs for this programme were separated by area, where possible, to account for location-specific differences in the implementation model.

We followed recommendations for costing programmes to manage child wasting by thoroughly documenting and reporting costs^(19,20)^. Table 2 outlines elements and considerations for both institutional and societal costs.

**Table 2.** Cost elements included

Cost element	Considerations and data needs
Institutional costs	
Programme staff time	Salary and time allocation data were collected for staff at all levels, including both support staff and implementing technical staff from both nutrition and FSL teams. We identified which categories of staff were not dedicated to the programme on a full-time basis. During staff allocation interviews, staff were probed about their time spent on activities related to the implementation of Tom Brown or Porridge Mum programmes *v*. other programmes or activities.
Government staff time	Salary and time allocation data for government staff were included, through costs paid by implementing partners, particularly for joint supportive supervision visits. These costs were extracted from implementing partners’ institutional accounting systems.
Local food costs	Costs of local food commodities were extracted from partners’ accounting databases and other documentation, either as unit costs and quantities from market assessments or, where this was unavailable, as a lump sum amount.
Storage and transportation	Including the operating costs of support/programme vehicles.
Vouchers and other FSL-related costs	Including the costs of voucher fees and other costs related to the reliance on the programme being embedded in the broader FSL programme (required for the Tom Brown programme to be implemented through vouchers). These costs were only included where and when vouchers and FSL-related costs were included in the programme design.
Office running costs	For national and field-level offices, including rent, utilities and security
Programme supplies	Non-food items (NFI) such as cooking utensils, storage containers, a floor mat, MUAC tapes and basic hygiene materials.
Stipends	Paid to community-based volunteers, including community nutrition mobilisers (CNM), lead mothers (LM) and field assistants (FA).
Other direct costs	Any not included above
Indirect costs	Including overhead attributable to the programmes
Societal costs	
Programme recipient time and resources	Time and money spent in preparing locally available foods (see Annex 3 in the supplementary materials for details on how these were estimated).
Kitchen provision	While no kitchen construction was necessary for Tom Brown, according to the Tom Brown programme staff, LM are required to use existing space within their homes to host Tom Brown group activities.
Local food vendor time and resources	Time, money spent to sell locally available foods or redeem vouchers, which could affect scalability.
Volunteer support (CNM, LM, FA and secretary/treasurer)	Support to activities provided in the community may have implications for scale-up, particularly the question of whether they receive an incentive. Because activities provided by CNM, LM and FA at the community level may have implications for scale-up, we collected additional information on these activities and the costs associated with them. Data collected from programme staff interviews provided additional information on the linkages between community-based volunteers, how their activities are aligned, which activities were compensated with incentives, which were contributed in-kind and what types of activities the volunteers are conducting in the communities. For any calculation of time spent, we used the same calculation method as described above for programme recipient time and resources. We also ensured that we did not double-count any contributions of LM when calculating programme recipient time and LM time since LM were also beneficiaries of the programme.
Value of in-kind storage space	As relevant for each intervention, particularly for PUI.
Government opportunity costs	Where the programme relies on existing government infrastructure for implementation such as joint supportive supervision. When government supervision was not included in institutional accounting systems, these costs were estimated separately and included in societal costs.

CNM, community nutrition mobilisers; CRS, Catholic Relief Services; FA, field assistant; FSL, food security and livelihoods; LM, lead mothers; MUAC, mid-upper arm circumference; PUI, Premiere Urgence Internationale; SCI, Save the Children International.

Costs were allocated to different cost categories for analysis according to their actual use or on the basis of reasonable assumptions or proxies for allocating shared indirect costs to specific programmes (i.e. cost driver rate or time allocation). Additional details on which costs were allocated to which category can be found in online supplementary material, Supplemental Fig. 1. These categories were developed by assessing accounting databases across the three partners. The final list of cost categories includes:

- **Supplementation**, including direct implementation costs such as stipends paid to community-based volunteers, food ingredients, NFI, costs of referrals and case finding and the cost of vouchers and associated fees. This also includes personnel costs specific to treatment, such as field assistants.
- **Community outreach**, including related printed materials, allowances and incentives and specific travel and personnel costs.
- **Storage and transportation**, such as the storage and transportation of food ingredients and NFI.
- **Training** attributable to the Tom Brown programmes, including trainer per diem, transportation reimbursement for participants and trainers, training materials, room hire and materials.
- **Supervision**, including personnel costs for nutrition officers and supervisory Tom Brown technical and programme staff, as well as relevant joint supervision conducted with government staff.
- **Management**, including broader programme management, MEAL and shared indirect and operating costs (including office rent).
- **Societal**, including the opportunity costs of participating group members and community-based volunteer labour (community nutrition mobilisers, lead mothers, field assistants and government staff, where appropriate). Also, the opportunity cost of the donated storage space to PUI’s Tom Brown programme.

### Data collection

Our data collection approach used existing accounting data and information from programme documentation and thirty retrospective staff interviews on resources used during programme development and implementation. Additionally, since programme approaches were embedded within existing nutrition and FSL structures, many capital cost investments (e.g. buildings and vehicles) not allocated by the partners as programme-specific investments were apportioned as daily transport hire or use costs.

Before data collection, the study team reviewed project documentation, including evaluations and enrolment information. During data collection, staff interviews focused on the main implementation offices in Borno state, with limited interviews in the country head offices in Abuja (see Annex 1 in the Supplemental Materials for interviews and questionnaires). Field data collection was conducted in April 2023 with follow-up via teleconference and email from April through August 2023.

Individual and group semi-structured interviews were conducted with programme staff to collect institutional and societal cost data (see supplementary materials for questionnaire). Staff were asked to estimate the programme-related recipient direct cost and time use. MEAL staff validated or collected data on the number of beneficiaries/children treated. Finance staff were interviewed to collect cost data to provide a better understanding of the finance systems and linkage of expenditures to programme activities. All qualitative interviews were confidential.

Qualitative interview transcripts were compiled and organised using a content analysis approach, with data systematically reviewed and extracted to identify themes. Quantitative accounting data were triangulated with qualitative interview data.

## Results

Table 3 summarises institutional and societal costs per category for the three programmes, presenting both total costs and cost per programme cycle. The costs of direct programme implementation (‘Supplementation’ category in Table 3) represented the largest overall cost category for nearly all programmes, ranging from 53 to 69 % of total costs. Limitations in accessing a comparable and full set of operating costs for the PUI programme likely resulted in an underestimation of total costs. Further, this underestimate of operating costs resulted in a higher proportion of costs allocated to direct supplementation (69 %) and lower management costs (4 %) compared with the other programmes. This finally resulted in a lower cost per child enrolled compared with the other programmes, despite the relatively low number of children enrolled.

**Table 3.** Institutional and societal expenditures by cost category, total cost and per cycle (USD)

Implementing partner	CRS								PUI		
Costs by cost category	CRS costs	Local partner costs	Total cost	CRS costs per group (8-week cycle)	Local partner costs per group (8-week cycle)	Societal costs per group (8-week cycle)	Total cost per group (8-week cycle)	% of sub-total/total	Total cost	Total cost per group (8-week cycle)	% of sub-total/total
Institutional costs											
Supplementation											
Direct Tom Brown costs (e.g. food and NFI for groups)	372 060	779 803	1 151 863	626	1313		1940	78·0 %	164 136	1026	79·0 %
Allowances and incentives for community-based volunteers	319 347		319 347	538			538	22·0 %	42 582	266	21·0 %
Case finding	151		151	0			0	0·01 %			
Referrals											
HR	9643·25		9643	16			16	0·65 %			
Sub-total supplementation			1 481 005				1493	62·0 %	206 719	1292	69·0 %
Community											
HR									10 360	65	100·0 %
Communications (printed materials, flyers, etc.)	84		84	0			0	100·0 %			
Other costs											
Sub-total community			84					0·0 %	10 360	65	3·0 %
Storage and transportation											
HR									3916	24	100·0 %
Storage	34 955		34 955	59			59	66·0 %			
Transport	17 993		17 993	30			30	34·0 %			
Sub-total storage and transportation			52 948				89	2·0 %	3916	24	1·0 %
Training											
HR	14 945		14 945	25			25	35·0 %			
Other costs	28 040		28 040	47			47	65·0 %	13 848	87	100·0 %
Sub-total training			42 985				72	2·0 %	13 848	87	5·0 %
Supervision											
HR	352 891		352 891	594			594	99·0 %	36 626	229	100·0 %
Government visits to communities											
Other costs	2227		2227	4			4	1·0 %			
Sub-total supervision			355 118				598	14·9 %	36 626		12·0 %
Management											
M&E	1077		1077	2			2	0·3 %	3675	23	32·0 %
Programme management	2126		2126	4			4	1·0 %			
Shared indirect costs (e.g. office costs, transport)	231 440		231 440	390			390	75·0 %			
HR	73 969		73 969	125			125	24·0 %	7649	48	68·0 %
Other costs											
Sub-total management			308 612				520	13·0 %	11 325	71	4·0 %
Societal costs											
Opportunity cost of community volunteers (LM, CNM and vendors)	134 979		134 979			227*	227	100·0 %	14 716†	92	100·0 %
Sub-total societal costs			134 979			227	227	5·7 %	14 716	92	5·0 %
Total	1 595 927	779 803	2 375 730	2460	1313	227	4000	100·0 %	297 510	1859	100·0 %

Implementing partner	SCI						
Costs by cost category	SCI Costs	Local partner Costs	Total Cost	SCI costs per group (10-week cycle)	Local partner costs per group (10-week cycle)	Total cost per group (10-week cycle)	% of sub-total/total
Institutional costs							
Supplementation							
Direct Tom Brown costs (e.g. food and NFI for groups)	11 767	492 677	504 444	37	1564	1601	56·7 %
Allowances and incentives for community-based volunteers		325 575	325 575		1034	1034	36·6 %
Case finding							
Referrals	2952		2952	9		9	0·3 %
HR		56 774	56 774		180	180	6·4 %
Sub-total supplementation			889 745			2825	53·4 %
Community							
HR	4119		4119	13		13	22·0 %
Communications (printed materials, flyers, etc.)							
Other costs	14 234	180	14 415	45	1	46	78·0 %
Sub-total community			18 534			59	1·1 %
Storage and transportation							
HR	13 697	24 437	38 134	43	78	121	32·0 %
Storage		3341	3341		11	11	3·0 %
Transport	91	75 831	75 922	0	241	241	65·0 %
Sub-total storage and transportation			117 397			373	7·0 %
Training							
HR							
Other costs	8820	1978	10 798	28	6	34	100·0 %
Sub-total training			10 798			34	0·6 %
Supervision							
HR	174 241	14 971	189 211	553	48	601	99·0 %
Government visits to communities	262	1375	1636	1	4	5	1·0 %
Other costs							
Sub-total supervision			190 847			606	11·5 %
Management							
M&E	28 580	1016	29 596	91	3	94	8·0 %
Programme management							
Shared indirect costs (e.g. office costs, transport)	151 878		151 878	482		482	43·0 %
HR	83 793	59 316	143 109	266	188	454	41·0 %
Other costs		26 262	26 262		83	83	7·0 %
Sub-total management			350 845			1114	21·0 %
Societal costs							
Opportunity cost of community volunteers (LM, CNM and vendors)	88 588^‡^		88 558	281		281	100·0 %
Sub-total societal costs			88 558			281	5·3 %
Total	582 992	1 083 731	1 666 723	1851	3440	529	100·0 %

* Weighted average from both CRS models.

† Includes donated storage space.

‡ Includes opportunity cost to FA.

The programme context influenced differences in storage and transportation costs. The three Tom Brown programmes operating in rural areas procured food ingredients in bulk and stored ingredients in different ways before delivering weekly to the lead mothers’ houses for food production. SCI stored the ingredients centrally at their Maiduguri office warehouse. CRS stored the ingredients in satellite offices throughout the rural coverage area. To avert supply chain breakdowns due to insecurity in Monguno, PUI stored the food ingredients locally at central warehouses before transporting them to the lead mothers’ houses, where activities took place. SCI’s decision to store items in their central Maiduguri warehouse as opposed to a more decentralised option resulted in higher programmatic costs and was a primary programmatic driver of their higher unit cost.

Our analysis of the CRS Tom Brown programme found that reliance on existing FSL structures for cash/voucher distribution had mixed cost implications. While the ability to leverage existing logistics and vendor networks helped reduce direct procurement and warehousing costs, these savings were partially offset by higher administrative and monitoring expenses due to their use of the cash/voucher model rather than direct procurement, as evidenced through staff interviews and cost data from SCI and PUI. Specifically, programme costs were influenced by the need for additional staff time for voucher redemption oversight, vendor transaction fees and quality assurance processes. The cost per recipient in the CRS Tom Brown programme was slightly higher in areas where vouchers were used, compared with locations where food ingredients were procured and distributed directly. These findings suggest that while cash/voucher models can enhance flexibility and market engagement, they may also introduce new cost considerations that require careful evaluation in future programme design. Additionally, the wide range in NFI costs was due to differences in procurement, storage and transportation practices across implementers and the scale of the programmes.

Table 4 presents the direct costs per Tom Brown group per programme cycle (8 or 10 weeks). Because these costs were estimated primarily from a combination of budget data and price lists, given the lack of disaggregation of partner accountancy data, we were only able to estimate the disaggregation of food and NFI costs at the per-group per-programme cycle level (Table 4).

**Table 4.** Direct costs per Tom Brown group per programme cycle (8 or 10 weeks)^*^

Cost type	CRS 8-week cycle		PUI 8-week cycle		SCI 10-week cycle	
	*n*	%	*n*	%	*n*	%
Institutional cost						
NFI/equipment per group	148·48	17·5 %	409·05	29·0 %	138·91	17·5 %
Food/ingredients	177·11	20·9 %	589·80	41·9 %	308·49	38·9 %
Transportation	30·29	3·6 %	–		15·60	2·0 %
Storage	58·85	6·9 %	24·47	1·7 %^§^	10·61	1·3 %
Preparation cost	202·82	23·9 %^†^	27·01	1·9%^||^	32·50	4·1 %^†^
LM stipends	4·55	0·5 %	129·63	9·2 %	5·20	0·7 %
Other stipends	–		136·51	9·7 %^¶^	–	
Societal cost						
Opportunity costs	227·24	26·8 %^‡^	91·98	6·5 %^**^	281·14	35·5 %^††^
Total	849·34		1408·50		792·45	

* These costs were estimated primarily from a combination of budget data and price lists, given the lack of disaggregation of partner accountancy data at this level. Percentages are calculated based on the cost per cost type divided by the total cost per group.

† Includes grinding, transport, firewood and water, along with cooking demonstrations.

‡ Includes opportunity costs for LM, recipient participating mothers and vendors and represents a weighted average from both CRS models (emergency/rural and peri-urban).

§ Excluding the donated storage space.

|| Includes grinding, transport, firewood and water.

¶ Includes CNM and FA stipends.

** Includes opportunity costs for LM, CNM, other recipient participant mothers, vendors and donated storage.

†† Includes opportunity costs for LM, CNM, FA and other recipient participating mothers, Ministry of Health staff and vendors.

Table 5 presents the opportunity costs to mothers participating in the programmes, in both USD and local currency (Naira). These are compared with the Nigerian monthly minimum wage. In two out of the three programmes, the value of time spent participating in this programme was 20 % of the monthly minimum wage, suggesting a relatively high time investment as measured in local wage rates.

**Table 5.** Opportunity cost per participating mother by implementing partner (USD and Naira)

Programme	CRS	PUI	SCI
Opportunity cost per month (USD)	$7·68	$2·92	$8·78
Opportunity cost per month (Naira)	5906	2247	6750
Percentage of Nigerian monthly minimum wage (30 000 Naira)	19·7 %	7·5 %	22·5 %

CRS, Catholic Relief Services, PUI, Premiere Urgence Internationale, SCI, Save the Children International.

Table 6 presents the societal costs for the three programmes, by cost element and stakeholder type, namely, volunteer cadre or programme recipient mothers. Time spent by lead mothers represented from 7·6 % up to 21·0 % of total measured societal costs for each programme, and food vendors in the CRS and PUI programmes represented 4·7–9·9 % of total societal costs. These findings suggest a relatively high burden on community volunteers to implement the programme.

**Table 6.** Total societal costs by cost element and type of volunteer cadre (USD)

	CRS			
	Peri-urban	Rural/emergency	PUI	SCI
Time of volunteer cadre^†‡§^				
CNM	–	–	$624 ($13·87) 3·8 %	$6045 ($35·35) 6·8 %
Lead mothers	$4719 ($22·91) 9·8 %	$8132 ($20·96) 9·4 %	$1248 ($7·8) 7·6 %	$18 557 ($58·91) 21·0 %
Field assistants	–	–	–	$7680 ($24·38) 8·7 %
Assistant participating mothers	$7130 ($17·31) 14·8 %	–	–	–
Other participating mothers	$31 633 ($15·36) 65·6 %	$71 501 ($18·43) 82·4 %	$10 296 ($5·26) 62·5 %	$55 283 ($21·94) 62·4 %
Food vendors	$4771 ($23·16) 9·9 %	$7093 ($18·28) 8·2 %	$781 ($4·88) 4·7 %	–
Government officials	–	–	–	$999 1·1 %
Value of donated resources^§^				
Donated storage	–	–	$1768 10·7 %	–

CNM, community nutrition mobiliser; CRS, Catholic Relief Services; PUI, Premiere Urgence Internationale; SCI, Save the Children International.

† These estimates are assumed to be underestimated as time spent by community-based volunteers and food vendors was estimated by interviews with programme staff and may not represent a complete set of time data.

‡ The opportunity cost per individual per volunteer cadre is represented in parentheses in each cell.

§ Percentages are calculated based on the cost per cadre divided by the total societal cost per programme.

Table 7 presents costs and cost-efficiency results for the three programmes and summarises key factors influencing cost per child, such as programme duration, implementation scale and modality (cash, vouchers or in-kind support). Total cost per child enrolled ranges from $155 to $184 per 8-week programme and $494 per 10-week programme. Monthly supplementation cost per programme recipient followed a similar pattern of costs at $5–8 per 8-week programme and $21 per 10-week programme.

**Table 7.** Overview of cost and cost-efficiency results by partner (USD)

Programme	CRS	%	PUI	%	SCI	%
Institutional cost	$2 240 750	94 %	$282 794	95 %	$1 578 165	95 %
Societal costs	$134 979	6 %	$14 716	5 %	$88 558	5 %
Total cost	$2 375 730		$297 510		$1 666 723	
Time period	23 months June 2021–April 2023		30 months January 2021–June 2023		23 months June 2021–April 2023	
Annualised total cost	$1 239 511		$119 004		$868 563	
Monthly cost	$103 293		$9917		$72 466	
Total no. children/beneficiaries enrolled	12 890		1920		3376	
Total cost per child/programme participant enrolled	$184		$155		$494	
Monthly supplementation cost per programme recipient	$8		$5		$21	
Key factors influencing cost per child/dosage	Emergency/rural + Peri-urban 8 weeks duration Food items procured in batches, stored in satellite offices and delivered to LM houses weekly. The peri-urban model has moved to cash vouchers. The longer implementation period allowed for attenuation of fixed costs.		Emergency/rural 8 weeks duration Food items procured in batches and stored in a central WFP storage facility (at no cost to the programme) and delivered to LM houses weekly. Costs are likely underestimated due to missing data on operational expenses.		Emergency/rural 10 weeks duration Food items procured and stored centrally at the SCI office/warehouse and delivered to LM houses weekly.	

CRS, Catholic Relief Services; LM, lead mothers; PUI, Premiere Urgence Internationale; SCI, Save the Children International; WFP, World Food Programme.

## Discussion

Despite the higher numbers of children suffering from moderate wasting globally, its management has not received the same level of attention or priority as severe wasting. This is due in part to the lower risk of mortality compared with severe wasting and the programmatic challenges presented by the larger caseload of moderately wasted children. Along with providing evidence to assist local implementers and stakeholders with programme planning, this costing study aims to impart information on assessing the scalability and replicability of the Tom Brown approaches and to contribute to the global evidence base on costs of different approaches for moderate wasting management.

In areas where coverage of TSFP for managing moderately wasted children is limited, Tom Brown may be considered a feasible alternative approach to TSFP in contexts where SFF are unavailable. The cost of these approaches will be an important factor in determining their feasibility and scalability. This cost and cost-efficiency analysis has highlighted several factors to consider when determining which approach, if any, is appropriate for the context. In addition to the total cost per child, the dosage-specific costs offer a complementary perspective, especially for comparing short-term interventions. Our findings will provide important considerations for governments, non-governmental organisations and funders aiming to integrate locally available food-based strategies into broader nutrition programmes globally.

When looking across Tom Brown programmes, we found that implementers generally used the same ingredients to produce the flour mixture, and the same NFI were used across programmes with slight variations. Although the basic kit was mostly the same across partners, the cost of providing those NFI per group varied by programme, ranging from $139 to $409. While the majority of the NFI provided to groups are mainly consistent across programmes, PUI also provides each group with a firewood stove, leading to an increased NFI cost ($409).

The existing literature on the cost implications of the management of moderate wasting is still limited and therefore might not accurately represent the full cost of such programmes or the potential variation across implementation contexts. To date, unit costs from analyses of TSFP programmes using SFF range from $83 to $100^(11,12)^. In the present analysis, there were clear differences in unit costs across the Tom Brown approaches. Two of the programmes (CRS and PUI) had a cost per child in line with and slightly higher than the aforementioned range of unit costs in the literature. The SCI programme yielded a unit cost more than double those figures and closer to a daily/weekly biscuit supplementation programme in Indonesia managing mildly and moderately wasted children, which ranged from $332 to $376^(4)^. The differences in unit costs across programmes were based on missing operating costs for PUI and differences in the analytical timeframe and associated implementation period, among other factors.

As illustrated by our analysis of CRS, the longer implementation period allowed for depreciation of initial programme start-up investments (e.g. equipment and staff capacity) and contributed to a lower cost per child compared with SCI. Differences in the numbers of beneficiaries reached by PUI compared with CRS and SCI contributed to differences in unit costs. Annualised costs and monthly supplementation costs per programme recipient represent more comparable cost figures.

Where possible, we isolated the costs of voucher/cash transfer programme components since scale-up may be planned in settings without these components. However, a detailed disaggregation of these costs was limited due to different partner accounting database structures. Yet when disaggregation was possible, it provided programmatic insights. For example, the cost of food ingredients for CRS purchased by lead mothers and participating mothers with their monthly vouchers accounted for 60 % of the CRS direct costs. The feasibility and scalability of CRS’ model of Tom Brown, or any similar programme with a voucher component, relies on an existing FSL infrastructure being in place (such as a programme recipient registration system and cash/electronic voucher distribution systems) and also benefits in efficiency from sharing that infrastructure.

Our analysis (Table 6) found that the use of cash/vouchers accounted for approximately 10 % of societal costs, due to the time participating recipient mothers spent redeeming vouchers for food ingredients and time spent by food vendors on redemption support and quality assurance. These factors have been measured in other studies, including a fresh food voucher programme in Pakistan, where food vendors’ high opportunity costs for time spent processing vouchers and preparing payment requests were not properly compensated with service fees, threatening their participation in the programme^(21)^. Cash and vouchers also potentially represent a significant institutional cost if an existing FSL programme is not already in place to support an existing supplementation or food delivery programme. Although vouchers reduced the need for direct food distribution, NFI such as cooking equipment still required storage and transportation, which contributed to overall costs. CRS’s procurement and logistics department was involved in managing the distribution of NFI and ensuring compliance with voucher redemption protocols. As such, the Tom Brown voucher model requires administrative oversight, monitoring and vendor coordination to manage voucher distribution and redemption. However, cash and/or voucher models may offer additional opportunities for sustainability by stimulating local demand, as vendors continue to stock and sell recipe ingredients if there is sustained interest from buyers after programme completion.

Locally available food-based approaches to manage moderate wasting presented a different set of opportunity costs, in terms of time requirements for participating households, compared with facility-based TSFP approaches. Facility-based approaches typically require a longer travel time compared with activities implemented closer to participants’ homes^(22)^. Our analysis showed that the opportunity costs of the Tom Brown interventions were substantial. Although as a proportion of total costs, societal costs were relatively small, ranging from 3 to 6 %, opportunity costs to an individual household may be significant. For example, compared with the minimum wage in Nigeria (30 000 Naira; $39 USD), the monthly opportunity cost per participating mother in conducting programme-related activities was valued at more than 10 % of the monthly minimum wage in nearly all programmes (Table 5). Our analysis further suggests that using cash/vouchers places a heavier burden on key community members such as food vendors, community workers and beneficiaries. Therefore, given that the core activities of these programmes relied on volunteer community members (i.e. active case finding, counselling of mothers and supervising groups’ food production), it seems more appropriate to provide a monthly wage (as CRS and SCI did) compared with a smaller stipend intended to cover only transportation costs (as did PUI). While providing wages may be difficult to sustain in some settings without external donor support and may complicate the transition to national health systems providing this type of service, it may also be difficult to sustain programmes relying entirely on volunteer effort with no remuneration.

This study is among the first to assess the costs of locally available food-based approaches to managing moderate wasting and presents the costs of three programmes using a unique service delivery approach, including an assessment of key societal costs. This study is expected to contribute to the limited evidence base for the cost of locally available food-based approaches to managing moderate wasting.

There are some limitations to this analysis. While this study’s disaggregation of costs has allowed for an assessment of cost drivers for each partner, this was limited in some cases due to retrospective assessment. Although this methodology is the most appropriate given the time and resource limitations for the implementation of this study, it relies on the availability and accuracy of the original cost and programmatic databases and cost recording systems, meaning that accuracy and reliability can vary across programmes^(23,24)^.

Institutional accounting systems are an important source of cost data for economic analysis. However, these databases have their challenges, resulting in limitations for research studies, including obtaining access to comparable datasets from different partners. This is a common but underexplored challenge in working with such data, particularly when comparing costs across different implementing organisations.

In our analysis, we noted several challenges in obtaining comparable sets of expenditure data from three partners with commensurate levels of detail to enable the separation of costs for specific programme activities. For example, while we planned to collect all institutional costs attributable to partner programmes, the PUI cost data were missing key operating costs (office costs and shared overhead costs at the Borno state and national level), which were included for the other programmes. While this resulted in an underestimation of and an inability to compare these costs with the others, the PUI programme analysis still contained a wealth of data on direct programmatic and societal costs. The full set of costs for the other three programmes enabled an understanding of these costing gaps.

Additionally, the structure of partners’ individual accounting databases sometimes created limitations in collecting comparable costs. For example, we were unable to isolate the costs related to the CRS cash/voucher component implemented in the peri-urban area in the direct supplementation cost category. Since the Tom Brown approaches were embedded in and relied on existing nutrition and FSL structures, assumptions were made about how to apportion those costs. This same challenge of finding similar costs as similar levels of disaggregation across partner databases further limited the analysis of food costs to the per-group and per-programme cycle level (Table 4).

Finally, our reliance on interviews with programme staff to estimate societal costs presented some limitations. Time spent by community-based volunteers and food vendors was estimated by interviews with programme staff and may be incomplete.

While this study provides useful information on programme costs and cost-efficiency, additional evidence on the relative effectiveness and cost-effectiveness of locally available food-based approaches is required to inform decisions on which approach is most appropriate in a given context. As nutrition stakeholders begin to put into practice the updated WHO guideline, especially as it relates to the management of lower-risk moderately wasted children, more studies should be conducted on intervention effectiveness and cost-effectiveness to inform decisions as to which approach (Tom Brown or TSFP) is most appropriate based on trade-offs between contextual appropriateness, effectiveness and costs at scale. Research should ensure the use of consistent methods, where possible, and the use of standard definitions of output and outcome indicators, as well as cost categories, to increase uptake and comparability of results^(19,20)^.

While our study provides important insights into the cost-efficiency of local food-based approaches like the Tom Brown programme, several factors must be considered when assessing the affordability and value for money of scaling these interventions. The affordability of such programmes will vary depending on local resource availability, existing health infrastructure and the degree to which donor and government funding can support their implementation. Additionally, future studies should explicitly examine the relationship between geographic coverage and cost-efficiency to better inform programme scalability.

In the Nigerian context, our findings suggest that the cost per programme recipient of the Tom Brown programme is comparable to, and in some cases lower than, traditional approaches using SFF such as ready-to-use supplementary foods. However, this cost-efficiency does not directly translate to cost-effectiveness, as our study did not evaluate health outcomes such as recovery rates, relapse rates or long-term nutritional improvements. To fully assess value for money, future research should focus on comparing the cost-effectiveness of local food-based interventions *v*. standard treatments for moderate and severe wasting. We recommend a multi-stakeholder funding approach. In Nigeria, a combination of national and regional government support, international donor contributions and community-based partnerships could ensure programme sustainability. Governments could integrate local food-based interventions into national nutrition and health strategies, while donors could prioritise funding for capacity-building and programme evaluation. Additionally, strengthening local markets through these programmes may create opportunities for community ownership and private sector engagement, contributing to long-term sustainability.

### Global policy implications

The results of this analysis are specific to the implementation context in northeast Nigeria, which is an ongoing emergency and food-insecure context. Implementation in this food-insecure context had specific implications for programme resource use. For example, all programmes provided food items to programme participants, the cost of which ranged from 20 to 42 % of direct costs per group (Table 4). In more food-secure contexts, mothers could purchase recipe ingredients with their own resources, with different implications for costs.

When considering scaling up or replicating these approaches, it is important to consider contextual factors that may impact coverage such as moderate wasting prevalence and population concentration. Security when travelling to a facility in certain regions and challenges with community-based programming in areas prone to displacement may also be a concern. Because they need to be established in each community, in some ways, Tom Brown programmes have higher upfront investments than a TSFP, which is already linked to an established facility covering multiple communities. However, as noted earlier, there are also trade-offs in opportunity costs of caregivers’ time to produce the flour/recipes *v*. travelling to the clinic to seek care. There is also the potentially higher opportunity cost of travelling to a health facility to find that treatment is unavailable because of low coverage or service delivery interruptions due to supply chain issues. Other factors contributing to costs, such as time spent in preparing the flour, may also have an impact on effectiveness due to time spent building community support, gaining lasting skills and receiving nutrition education and counselling. Further research is needed to assess the full set of benefits relative to costs.

This study highlights the complexities that arise when standardised cost accounting methods are not uniformly applied by implementing agencies, which complicates the assessment of true costs across different intervention types. To address this, we recommend that the Global Nutrition Cluster take the lead in developing a standardised costing framework for nutrition interventions in humanitarian settings. This would enable more consistent cost comparisons across programmes and contexts, facilitating better decision-making by stakeholders.

Despite differences in costing approaches, our findings suggest that local food-based approaches were, depending on the implementer, cost-comparable to the standard TSFP model. This is a critical insight for national and regional governments, as it highlights the potential for local food-based interventions to have similar costs to TSFP and also offer the opportunity to strengthen local markets, promote caregiver autonomy in the management of moderate wasting, along with permitting more points of contact for social and behaviour change communication, which could be advantageous.

However, the variance in cost estimates was influenced both by differences in how costs were captured and by the actual implementation costs incurred by different organisations. This suggests that if cost savings are the primary motivation for shifting to local food-based approaches, further investigation is warranted to understand the drivers of cost variability. We recommend that funders and non-governmental organisations prioritise additional research to evaluate the cost-effectiveness and scalability of these approaches, considering both economic and social impacts.

### Conclusions

This costing study in Nigeria is an important step towards building the economic evidence base for the use of locally available foods in the management of moderate wasting and is in accordance with the recently released WHO guideline, which includes updated guidance on the management of moderate wasting^(3)^. The guideline also places an emphasis on the use of nutrient-dense foods to support recovery, including locally available foods typically consumed by households. Factors that place some moderately wasted children at higher risk of mortality are also discussed along with recommendations that these children be prioritised to receive SFF through the health system. Children not meeting one or more of these risk factors can be supplemented with locally available foods^(3)^. Given evidence from this study, the cost per programme recipient to manage moderate wasting with locally available foods potentially would be similar to or higher than TSFP and other options to manage moderate wasting but would represent a feasible and locally acceptable option in the absence of funding and availability of SFF, allow for more children with moderate wasting to be reached and potentially bring other benefits in the form of community cohesion and prevention of further moderate wasting. This suggests potential for scaling up programmes like Tom Brown as countries adapt their waste management protocols to align with WHO guidance, particularly in contexts where local food-based interventions are feasible and cost-efficient.

## Supporting information

Gobin et al. supplementary material Gobin et al. supplementary material

## Supplementary material

For supplementary material accompanying this paper, visit https://doi.org/10.1017/S1368980025101213.
