# Supplementary material for: The Cost of Managing Moderate Wasting Using Local Foods: Evidence from Three Interventions in Northeast Nigeria
Source: Public Health Nutr. 2025 Sep 30;28(1):e181. doi: 10.1017/S1368980025101213 (PMC12722097; doi:10.1017/S1368980025101213)

Table of Contents

[Annex 1: Data Collection Tool 2](#_Toc201221428)

[Annex 2: Program Descriptions 13](#_Toc201221429)

[Catholic Relief Services (CRS) 13](#_Toc201221430)

[Premiere Urgence Internationale (PUI) 13](#_Toc201221431)

[Save the Children International (SCI) 14](#_Toc201221432)

[Annex 3: Details on Beneficiary Time and Resource Calculations 15](#_Toc201221433)

[Supplemental Figure 16](#_Toc201221434)

# Annex 1: Data Collection Tool

MAM Treatment with Locally Available Food-Based Approaches (2023)

P**lease read the consent form and ask for the respondent's approval before starting the interview.**

| **1.** Name of Interviewer |  |
| --- | --- |
| **2.** Date of interview (MM/DD/YYYY) |  |
| **3.** Questionnaire Number |  |
| **4.** Respondent ID |  |
| **5.** Partner Organization |  |
| **6.** Community Name/Code |  |
| **7.** Name of Respondent |  |
| **8.** Title of Respondent |  |

*Questionnaire is mainly directed toward program staff (with the potential to include some food vendors if logistically feasible).*

Complete the answer(s) in the appropriate section below, do not duplicate.

| **Program Staff Costs Questions** |  |
| --- | --- |
| **9.** Total number of staff that have contributed to the implementation of the program (procure a list for additional probing) |  |
| **10.** Which staff work on the program 100%? |  |
| **11.** Probe for additional details of role of specific staff who may require time allocation interviews and for those who may only work a proportion of their time on the program |  |
| **12.** Probe additional questions to understand the cost data outputs and how to tie staff time to cost categories (i.e., training, supervision, TA, etc.) |  |
| **13.** What is the role of the government in the program? Do government ministries provide any resources necessary for implementation? If so, what are these: (i.e.,  1. staff time  2. direct inputs  3. indirect contributions,  4. those costs that need to be considered for scale-up)? |  |
| **14.** Where can we see food storage costs in the cost data? |  |
| **14.b** If food storage costs are “off-budget” (or otherwise not found in the program cost data), what are the resources required to store the food?  **(NB:** Collect information here about building rent, size of building, space used for food storage (%), cost of guards, etc.). |  |

| **Beneficiary time and resources** |  |
| --- | --- |
| **15.** List all activities beneficiaries participated in during the preparation of locally-based foods (Tom Brown or Porridge Mums).    For example:  1.Washing food  2. Husking  3. Grinding and mixing  4. Collecting clean water  5. Counselling  6. Any others? |  |
| **16.** List time spent by beneficiaries for each activity in Q9 |  |
| **17.** What % of beneficiaries pay to have grinding done versus those who do the grinding themselves? |  |
| **17.b** What is the cost of grinding if it is done by beneficiaries themselves? |  |
| **17.c** What is the cost of grinding if it was paid for with cash by beneficiaries? |  |
| **18.** How much firewood is used per week, or what is the cost of electricity used per week in cooking locally-based foods? (local currency) |  |
| **19.** Time spent by beneficiary in fetching firewood per week. |  |

| **Kitchen provision and construction** | *Kitchen construction questions are relevant for Porridge Mums partners* |
| --- | --- |
| **20.** What is the total cost of the kitchen construction? |  |
| **20.b** For Tom Brown partners, was any kitchen adaptation/construction done to the lead mothers’ homes? (including equipment). If yes, please list and describe the costs associated with. |  |
| **21.** How much or percentage of the kitchen cost was paid/contributed by local communities? |  |
| **22.** List all the resources that were used for the construction of kitchen. How many of these were paid/contributed by the community versus a partner organization? |  |
| **23.** List all activities performed by local community members and groups constructing kitchen for preparation of locally-based foods. |  |
| **24.** How much time was spent on each activity listed Q23 *(List those involved, and the # of days/hours spent)* |  |
| **25.** The value of the resources that were provided by the community? *(include the value of resources listed in Q22 and the value of any community time donated)* |  |

| **Local food vendor time and resources** |  |
| --- | --- |
| **26.** What type of food vendor is used in the program? |  |
| **27.** Are food vendors paid service fees or other costs? If so, please list and describe these and how frequently are they paid. |  |
| **28.** How much time is spent (in minutes) by food vendors in selling locally available foods and ingredients. Activities may include processing voucher requests, preparing payment requests, and other transaction. |  |
| **29.** How many times do food vendors vend to beneficiaries per week? |  |

| **Local Foods Production** |  |
| --- | --- |
| **30.** List all ingredients (in units i.e., metric tons, bags, pans, as appropriate) used in the preparation of 1 batch of the selected locally-based foods |  |
| **31.** Provide cost for each ingredient listed in Q30 for preparing locally-based foods (in local currency) |  |
| **32.** Was the local food prepared by beneficiaries or by the CNM/lead mothers? |  |
| **33.** How many volunteers helped in the implementation of locally-based foods for treatment of MAM? |  |
| **34.** What type of volunteers are involved in the production of the locally-based foods? *(CNMs, Lead Mothers, any others?)* |  |
| **35.** What is the educational background of CNM/LMs? Other volunteers? |  |
| **36.** List all activities volunteers provided in support of the implementation of locally-based foods    For example:  1.Training of beneficiaries  2. Kitchen construction  3. Transportation of ingredients  4. Food preparation e.g., Tom Brown  5. Transportation of finished locally-based foods to beneficiaries  6. Any others? | \|  \| CNM \| LM \| … \|  \|  \| \| --- \| --- \| --- \| --- \| --- \| --- \| \| Training BNFs \|  \|  \|  \|  \|  \| \| Kitchen construction \|  \|  \|  \|  \|  \| \| Transport ingredients \|  \|  \|  \|  \|  \| \| Food preparation \|  \|  \|  \|  \|  \| \| Transport locally-based foods \|  \|  \|  \|  \|  \| \| … \|  \|  \|  \|  \|  \| \|  \|  \|  \|  \|  \|  \| |
| **37.** List time spent by volunteers for each activity in Q36 in support of implementing LPF *(List those involved, and the # of days/hours spent)* | \|  \| CNM \| LM \| … \|  \|  \| \| --- \| --- \| --- \| --- \| --- \| --- \| \| Training BNFs \|  \|  \|  \|  \|  \| \| Kitchen construction \|  \|  \|  \|  \|  \| \| Transport ingredients \|  \|  \|  \|  \|  \| \| Food preparation \|  \|  \|  \|  \|  \| \| Transport locally-based foods \|  \|  \|  \|  \|  \| \| … \|  \|  \|  \|  \|  \| \|  \|  \|  \|  \|  \|  \| |

| **CNM and lead mother support** |  |
| --- | --- |
| **38.** List all activities CNMs/lead mothers performed specifically in the food preparation of locally-based foods (Tom Brown or Porridge Mums) and the time spent by CNMS/lead mothers for each activity. *(List the # of days/hours spent per batch)*    For example:  1.Washing food  2. Husking  3. Grinding and mixing  4. Collecting clean water  5. Counseling  6. | \|  \| CNM \| LM \| … \| \| --- \| --- \| --- \| --- \| \| Washing food \|  \|  \|  \| \| Husking \|  \|  \|  \| \| Grinding and mixing \|  \|  \|  \| \| Collecting clean water \|  \|  \|  \| \| Counseling \|  \|  \|  \| \| … \|  \|  \|  \| \|  \|  \|  \|  \| |
| **39.** Do CNMs receive an incentive for their work on the program? If yes, what is the incentive? |  |
| **40.** Do CNMs incur any out-of-pocket costs (i.e., for transportation or anything else). If so, how much and what was the frequency. |  |
| **41.** Do lead mothers receive an incentive for their work on the program? If yes, what is the incentive? |  |
| **42.** Do lead mothers incur any out-of-pocket costs (i.e., for transportation or anything else). If so, how much and what was the frequency. |  |
| **43.** Cost of grinding if it was paid for by CNM/lead mother |  |
| **44.** Cost of electricity or firewood use per week in cooking locally-based foods (local currency) if paid for by CNM/lead mother |  |
| **45.** Time spent by CNM/lead mother in fetching firewood per week. |  |

**Number of Interviews Conducted per Program**

| **Program** | **CRS** | **PUI** | **SCI** |
| --- | --- | --- | --- |
| No. Interviews (Individual and Group) | 12 | 5 | 5 |

# Annex 2: Program Descriptions

### Catholic Relief Services (CRS)

**Program Description**

CRS has a long history of including Tom Brown as part of its nutrition activities in Nigeria as part of its work to support orphans and vulnerable children and broader food security and nutrition efforts. The USAID-funded Feed the Future Nigeria livelihoods projects and the Sustainable Mechanisms for Improving Livelihoods and Household Empowerment (SMILE) project, implemented from 2013-2018, provided caregivers with the Tom Brown flour to make porridge for moderately wasted children at home. In 2018, CRS Nigeria decided to expand the use of Tom Brown into a humanitarian context to close an identified gap in services for children with moderate wasting. CRS and its partners Justice, Development, and Peace Commission (JDPC) and North East Youth Initiative Forum (NEYIF) piloted and scaled up the Tom Brown supplementary feeding program in six LGAs across Borno and Yobe states. JDPC and NEYIF continue to provide support to CRS to implement Tom Brown programs, including all of the direct community-level implementation within defined LGA catchment areas.

CRS implementation of Tom Brown begins with selection of LMs to facilitate the groups, and advocacy and sensitization activities to inform caregivers about the mass MUAC screening of children aged 6-59 months. All moderately wasted children are enrolled in a Tom Brown group, and severely wasted children are referred to the nearest outpatient treatment program (OTP). Sensitization activities are conducted by CNMs. CRS used slightly different program models in its rural and peri-urban implementation areas. In peri-urban areas, CRS has shifted to using cash and vouchers rather than in-kind food items to produce the Tom Brown flour. For peri-urban areas, CRS also selects two assistant beneficiary mothers who support the LMs in their duties, including purchasing the local food items from vendors.

**Ingredients and Materials Provided per Group**

Ingredients (weekly amounts per group): Soya (8kg), millet (8kg), sorghum (8kg), ground nut (2.7kg), cloves (.5kg)

Materials: Lead mother kitchen kits and equipment and MUAC tape

### Premiere Urgence Internationale (PUI)

**Program Description**

PUI implements a full package of nutrition activities in North East Nigeria, including support to inpatient and outpatient treatment of severe wasting in Maiduguri and Monguno. In Monguno, PUI also supports the management of moderate wasting using the Tom Brown approach. Enrollment of children for Tom Brown is done by CNMs through routine MUAC assessments.

PUI’s transactional cost data does not include any storage costs for food ingredients because the program relies on storage space donated by WFP. The value of this storage space was estimated based on the size of the space, the amount of space being utilized by the Tom Brown ingredients, and the value of comparable storage facilities in the area.

**Ingredients and Materials per Group**

Ingredients (weekly amounts per group): Soya beans (9kg), millet (9kg), sorghum (9kg), ground nut (3kg)

Materials: Rubber bowl, frying pan, trays, measuring containers, firewood stove, airtight containers, cups with covers, matt, colander, and frying spoon. PUI also provides hygiene kits and NFI such as soaps to beneficiary mothers during graduation.

### Save the Children International (SCI)

**Program Description**

As part of SCI’s integrated program, “Integrated lifesaving assistance for conflict-affected households in Borno, Nigeria”, which began in July 2021, SCI is implementing nutrition activities including both preventive (maternal infant and young child nutrition) and curative (CMAM) components. The CMAM components involve routine MUAC screening and referral of wasted children under five, management of severe wasting in inpatient and outpatient treatment sites, the Tom Brown approach to manage moderate wasting, and Community Management of At-Risk Mothers And Infants (CMAMI) services.

**Ingredients and Materials Provided per Group**

Ingredients (weekly amounts per group): Soya (8kg), millet (8kg), sorghum (8kg), ground nut (2.7kg), cloves (.5kg),

Materials: Mat, rubber cups, airtight containers, pots with cover, frying pan, perforated frying spoons, ladle, colander, plastic bags, empty sack, local trays, rubber bowls, hand washstand, liquid hand wash, measuring containers, rubber bucket, and masks.

#

# Annex 3: Details on Beneficiary Time and Resource Calculations

Beneficiary time and resource calculations in the study included the following items:

Time and money spent in preparing locally available foods.

- 1. Program staff estimates of the time required by beneficiaries per week.
     - For Tom Brown: each week 12-15 caregivers plus one LM take the first 3 to 4 days of each week to prepare Tom Brown and distribute take-home rations. We assessed how long they spend on average during each of these days for grinding the grain, food preparation, collecting clean water, giving/receiving counseling, and other relevant activities.
  2. A local daily wage estimate was used to value beneficiary time. We have used an estimate of the most relevant local daily wage as a shadow wage for program beneficiaries in the calculations. A shadow wage is an estimation of the economic value of the resource when the market value is unavailable. For volunteer roles (e.g., LM, FA, secretary/treasurer, and in some cases CMNs) we assumed the published national minimum wage and for government staff, where relevant, we assumed the equivalent of a mid-range field-based MOH supervisor.
  3. Costs associated with using the grinding mill and/or time cost to walk to the mill vary by location (some interviewees reported long distances required to walk to the mill).

# Supplemental Figure

**Figure 1. Allocation of program costs to cost centers for analysis**


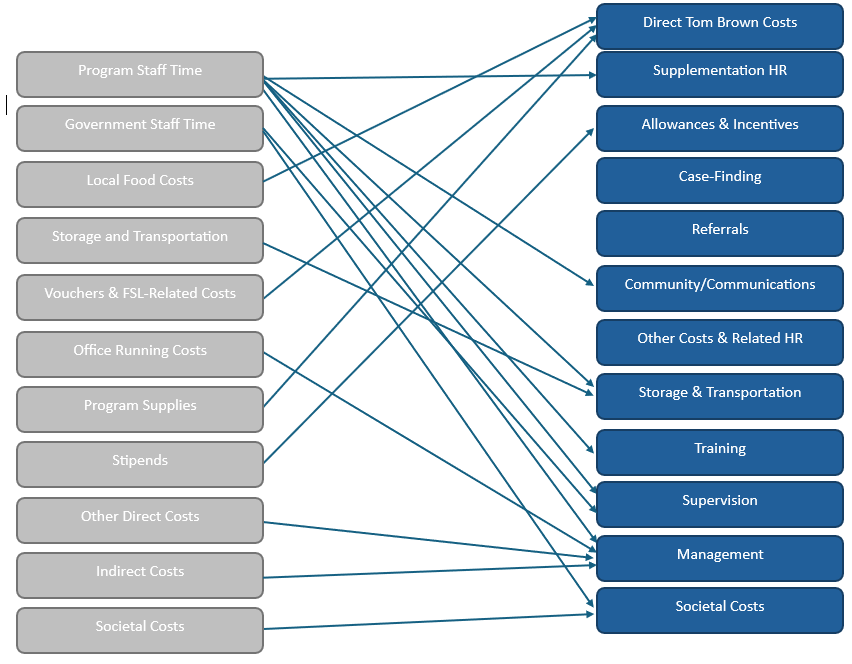

Supplement: Gobin et al. supplementary material [file S1368980025101213sup001.docx]
